# Bioassays for TSH Receptor Autoantibodies, from FRTL-5 Cells to TSH Receptor–LH/CG Receptor Chimeras: The Contribution of Leonard D. Kohn

**DOI:** 10.3389/fendo.2016.00103

**Published:** 2016-07-25

**Authors:** Cesidio Giuliani, Motoyasu Saji, Ines Bucci, Giorgio Napolitano

**Affiliations:** ^1^Unit of Endocrinology, Department of Medicine and Sciences of Aging, Ce.S.I.-Me.T., University of Chieti–Pescara, Chieti, Italy; ^2^Department of Internal Medicine, Division of Endocrinology, Diabetes and Metabolism, The Ohio State University, Columbus, OH, USA

**Keywords:** TSH receptor bioassay, FRTL-5 cells, Graves’ disease, TSHR autoantibodies, chimera

## Abstract

Since the discovery 60 years ago of the “long-acting thyroid stimulator” by Adams and Purves, great progress has been made in the detection of thyroid-stimulating hormone (TSH) receptor (TSHR) autoantibodies (TRAbs) in Graves’ disease. Today, commercial assays are available that can detect TRAbs with high accuracy and provide diagnostic and prognostic evaluation of patients with Graves’ disease. The present review focuses on the development of TRAbs bioassays, and particularly on the role that Leonard D. Kohn had in this. Indeed, 30 years ago, the Kohn group developed a bioassay based on the use of FRTL-5 cells that was characterized by high reproducibility, feasibility, and diagnostic accuracy. Using this FRTL-5 bioassay, Kohn and his colleagues were the first to develop monoclonal antibodies (moAbs) against the TSHR. Furthermore, they demonstrated the multifaceted functional nature of TRAbs in patients with Graves’ disease, with the identification of stimulating and blocking TRAbs, and even antibodies that activated pathways other than cAMP. After the cloning of the TSHR, the Kohn laboratory constructed human TSHR–rat luteinizing hormone/chorionic gonadotropin receptor chimeras. This paved the way to a new bioassay based on the use of non-thyroid cells transfected with the Mc4 chimera. The new Mc4 bioassay is characterized by high diagnostic and prognostic accuracy, greater than for other assays. The availability of a commercial kit based on the Mc4 chimera is spreading the use of this assay worldwide, indicating its benefits for these patients with Graves’ disease. This review also describes the main contributions made by other researchers in TSHR molecular biology and TRAbs assay, especially with the development of highly potent moAbs. A comparison of the diagnostic accuracies of the main TRAbs assays, as both immunoassays and bioassays, is also provided.

## Introduction

Thyroid-stimulating hormone (TSH) receptor (TSHR) autoantibodies (TRAbs) are the pathogenic hallmark of Graves’ disease. They are detected in nearly all untreated patients with Graves’ disease and are responsible for the pathological features of this disease (i.e., stimulation of thyroid growth and function, onset of orbitopathy, and/or dermopathy) (1). Several varieties of TRAbs have been described: stimulating (TSAbs), blocking (TBAbs), and neutral (N-TRAbs). Their relative concentrations define the clinical picture and the progression of Graves’ disease. Indeed, quantitation of TRAbs is of clinical use not only to confirm the diagnosis of Graves’ disease but also to predict the evolution of the disease and its complications, such as orbitopathy (2, 3). Furthermore, TBAbs are involved in the pathogenesis of hypothyroidism in the atrophic form of Hashimoto’s thyroiditis (4).

**Figure 1:**
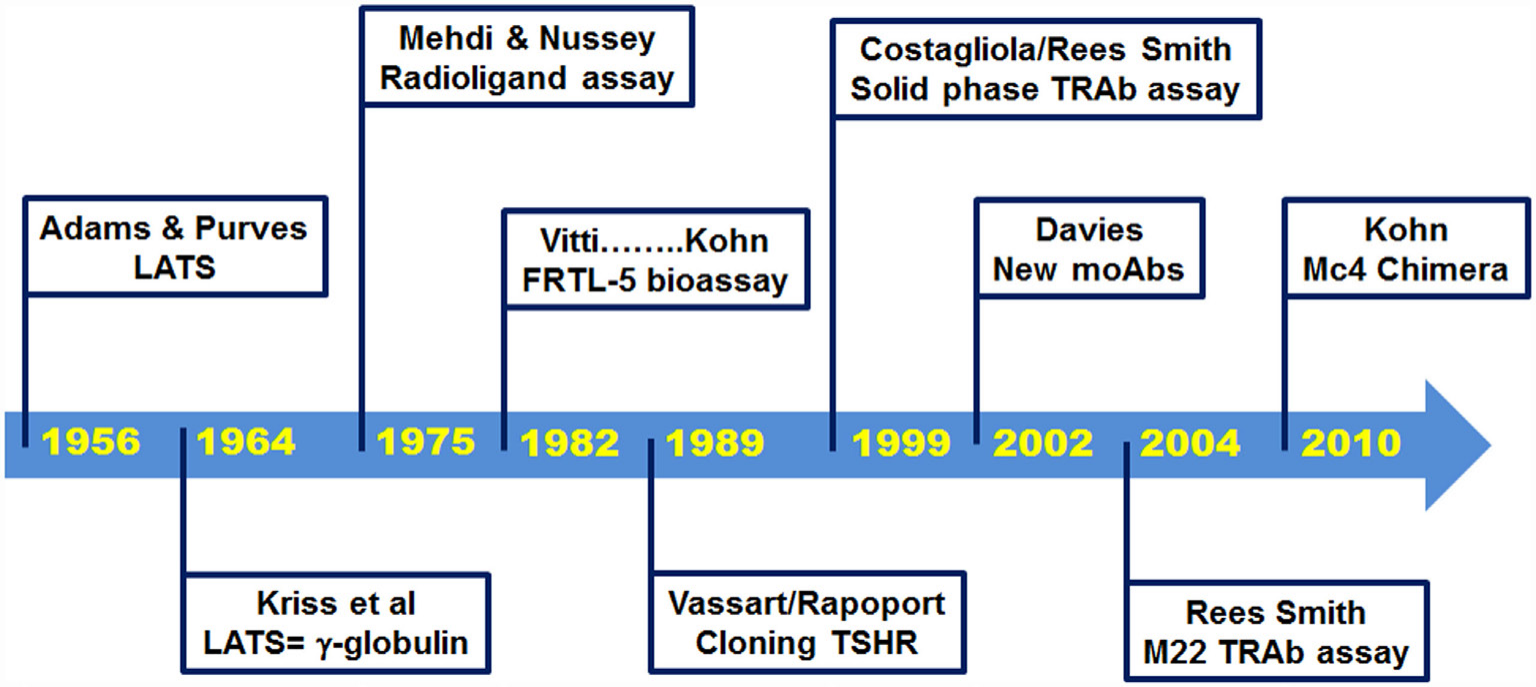
**Milestones in history of TSH receptor antibody assays**.

Since the discovery by Adams and Purves in 1956, of a thyroid-stimulating factor in the serum of some thyrotoxic patients (5), remarkable progress has been made in the knowledge of the biological properties of TRAbs. Furthermore, very sensitive assays are now commercially available to detect TRAbs (Figure 1). The purpose of this article is to review the development of these TRAbs bioassays, with a focus on the contributions made here by the late Dr. Leonard D. Kohn.

## Historical Background

In 1956, Adams and Purves noted that sera from thyrotoxic patients induced abnormal prolonged responses in their TSH bioassay that used guinea pigs (5, 6). They initially named the unknown substance that was responsible for this effect as the “abnormal thyroid stimulator,” and then later as the “long-acting thyroid stimulator” (LATS) (6, 7). Soon after its discovery, it became apparent that this LATS was distinct from endogenous TSH and that it was not produced by the pituitary (7). In 1964, LATS was identified as a protein with the biological characteristics of an antibody (8), and further studies unequivocally demonstrated its identification with immunoglobulin G (IgG) (9, 10).

The early *in vivo* bioassays to detect LATS were performed using guinea pigs or mice, but these were of little use in clinical practice as they were troublesome and had very low sensitivity. Indeed, 30–40% of patients with Graves’ disease were negative with these assays (11). A significant breakthrough was then made in 1975, with the development of a radioligand receptor assay, which evaluated the inhibition by the sera from patients with Graves’ disease of the binding of radiolabeled TSH to human thyroid membranes *in vitro* (12). However, this assay was still burdened by low accuracy. Further improvements to the method were provided by the use of the partially purified TSHR instead of thyroid membranes and biologically active radiolabeled TSH. This thus led to the development of a reproducible and accurate radioligand assay some years later (13, 14). This assay has been defined as a liquid phase first-generation immunoassay, and it was widely used for the next 20 years. It had a specificity of 99.2% (range, 97.5–100%) and a sensitivity of 79.8% (range, 52–94%) (15).

In parallel with the development of the radioligand receptor assay, there was also an improvement in the bioassay methods, with the replacement of the *in vivo* assay with *in vitro* techniques, such as the use of thyroid slices or thyroid primary cell cultures (16). A further fundamental advance was obtained with the development of FRTL-5 cells, a non-transformed cell line of rat thyroid epithelial cells in continuous culture (17). Indeed, the Kohn laboratory at the National Institutes of Health in Bethesda used these FRTL-5 cells to set up an accurate assay for the measurement of TSAbs, which provided greater convenience and reproducibility compared to other bioassays (18–20). From that time, FRTL-5 cells became the preferred tool for TRAbs bioassays for more than 10 years, and as discussed below, they were fundamental to the determination and quantification of the functional properties of TRAbs.

## The FRTL-5 Bioassay

FRTL-5 cells are a cell line that can be grown in continuous culture and that retains all of the properties of normal thyroid cells. Soon after their development, the Kohn group described the optimal conditions to measure TSAbs using FRTL-5 cells (18, 19). The assay was based on the ability of purified IgG preparations to induce cAMP production. Removal of TSH from the culture medium resulted in an enhanced response to acute stimulation by TSH and TSAbs. This assay showed a specificity of 97.6% and a sensitivity of 90.4%, thus providing a sensitivity that exceeded that of the liquid phase first-generation immunoassay (19, 21). The assay method was patented (22), and this paved the way to the commercial availability of the bioassay, and to its spread. Of note, all of the royalties associated with this patent were dispensed in the forms of grants to international researchers in the field of thyroidology. A further improvement in the feasibility of this test was provided with the direct use of the patient sera, rather than the purified IgG (23).

This FRTL-5 bioassay was not only important for diagnostic purposes but also a fundamental tool in the characterization of the functional properties of TRAbs and the understanding of their pathogenic role in Graves’ disease. The Kohn laboratory was particularly active in pursuing this. Indeed, it was Kohn and his colleagues who first developed monoclonal antibodies (moAbs) against TSHR, and they used the FRTL-5 cells to evaluate their functional properties (24–26). The generation of moAbs from lymphocytes of patients with Graves’ disease was also of significance, as this demonstrated the multifaceted functional nature of TRAbs, with some stimulating and others blocking the receptor activity (25, 26). These data were of great importance for the confirmation of TBAbs in Graves’ disease, as had been postulated previously (27, 28).

**Figure 2:**
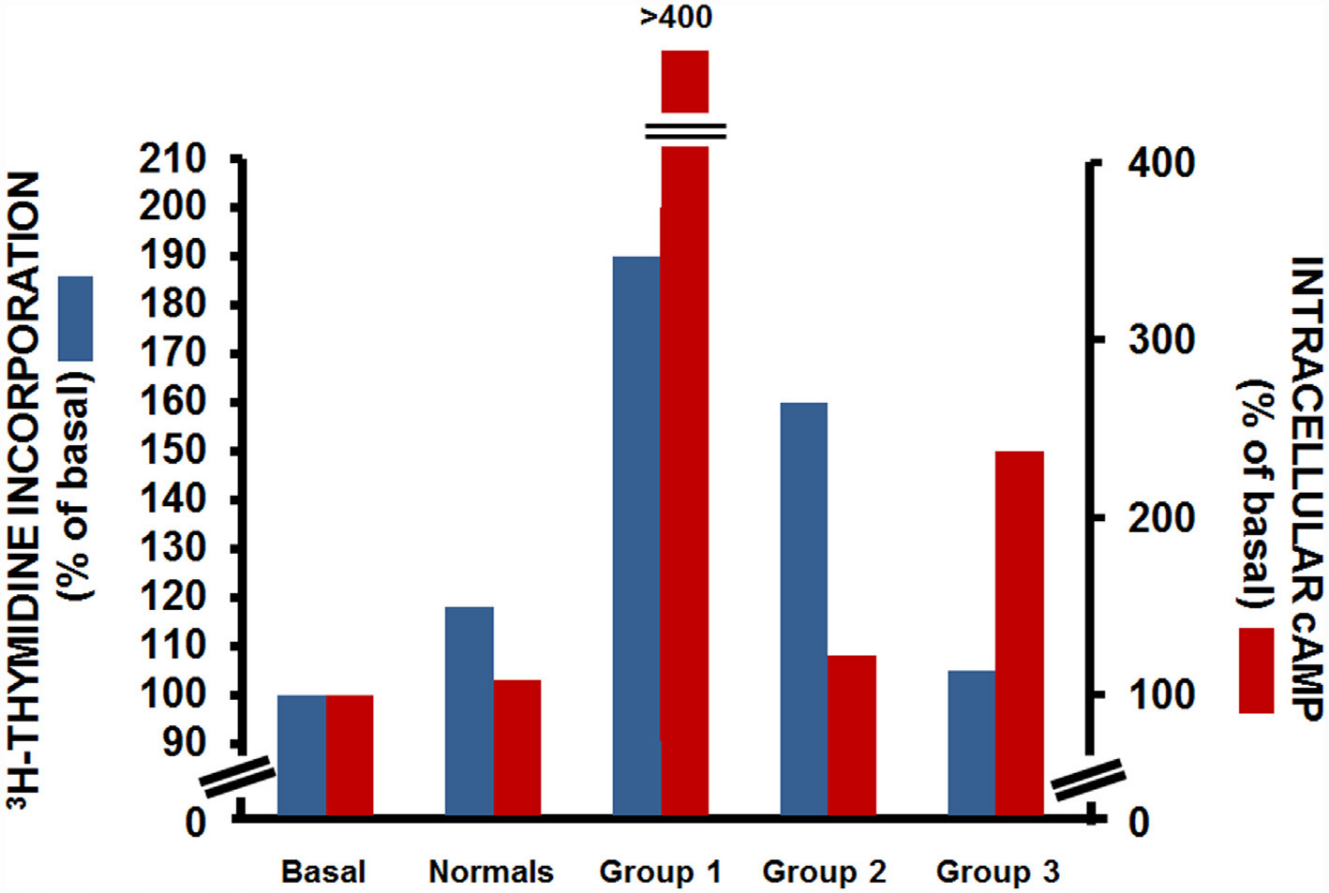
**Effects of IgG from patients with Graves’ disease on [^3^H]-thyimidine incorporation (blue) and intracellular cAMP production (red) in FRTL-5 cells**. Data from three representative patients with Graves’ disease for each group. Basal, cells with no treatment; normals, cells incubated with a pooled sample of IgG from 13 non-Graves’ individuals [Data are from Ref. (29)].

The use of the FRTL-5 cells also provided the possibility to further study the functional heterogeneity of TRAbs, as they allowed the separate assessment of the effects of an individual IgG on two distinct cellular activities: those of the production of cAMP and of cell growth. Indeed, Kohn and colleagues performed both cAMP assays and thymidine-incorporation assays in cells incubated with sera from patients with Graves’ disease. Through this, they demonstrated that these patients with Graves’ disease fell into one of three groups (Figure 2): those where the IgGs had strong cAMP-stimulating activity together with strong growth-promoting activity (group 1); those where the IgGs had strong growth-promoting activity, but little or no cAMP-stimulating activity (group 2); and those where the IgGs had strong cAMP-stimulating activity, but low growth-promoting activity (group 3) (29). This study demonstrated the separate and distinct effects of TRAbs on cAMP production and cell growth, which suggested that other transduction mechanisms as well as cAMP might be involved in their interactions with TSHR. This assumption was later confirmed by several studies, most of which were performed in the Kohn laboratory, which showed that the growth and function of thyroid cells were dependent on the ability of TSH to activate not only cAMP signaling but also other signaling pathways, such as those of phospholipase C and phospholipase A_2_/arachidonic acid (30, 31). A further confirmation came from studies, which showed that a subpopulation of IgGs from patients with Graves’ disease activated the phospholipase A_2_ pathway without affecting the cAMP signal (32, 33). These studies were fundamental to the correlation of the clinical heterogeneity of Graves’ disease with its pathogenesis.

Today, the heterogeneity of TRAbs is well recognized, and in addition to the classical TSAbs and TBAbs, which act as TSH agonists and antagonists, respectively, other forms of TRAbs have been described, in terms of the neutral antibodies (N)-TRAbs (Table 1). The N-TRAbs are so called because their binding to TSHR does not influence the binding of TSH and the cAMP levels, although they can activate other signaling cascades (1, 34, 35). Moreover, some of the antibodies that have been regarded as TBAbs have shown some growth-promoting activity independent of cAMP signaling (36).

**Table 1:** **Summary of the functional characteristics of TRAbs**.

Antibody	Effect on TSH binding	Effect on cAMP levels	Interference with cAMP-independent signaling
Stimulating	Inhibition	Increase	Yes
Blocking	Inhibition	Inhibition	Yes
Neutral	No effect	No effect	Yes

## The Cloning of the TSH Receptor

A major breakthrough in thyroid research arrived with the cloning of TSHR in 1989 (37–39). The cloning allowed the use of the recombinant human (rh-)TSHR, both for the radioligand receptor assay and the bioassay (40, 41). This led to an increase in the sensitivity of the radioligand receptor assay to 96% (41), which was higher than that of the FRTL-5 bioassay. The cloning also improved the feasibility of the use of the bioassay, as it was possible to transfect rh-TSHR into non-thyroid cell lines that were characterized by simpler culture conditions than the FRTL-5 cells (42, 43). The new transfected rh-TSHR bioassay was also characterized by better sensitivity than the FRTL-5 bioassay. Indeed, a comparative study performed using purified IgGs from 58 patients with Graves’ disease showed that a bioassay based on Chinese hamster ovary (CHO) cells transfected with rh-TSHR had a higher sensitivity than the FRTL-5 bioassay (93 vs. 75.8%, respectively) (42). These data were confirmed by an independent study that showed a similar sensitivity for these two bioassays (92.2 and 74.5%, respectively) (43).

**Figure 3:**
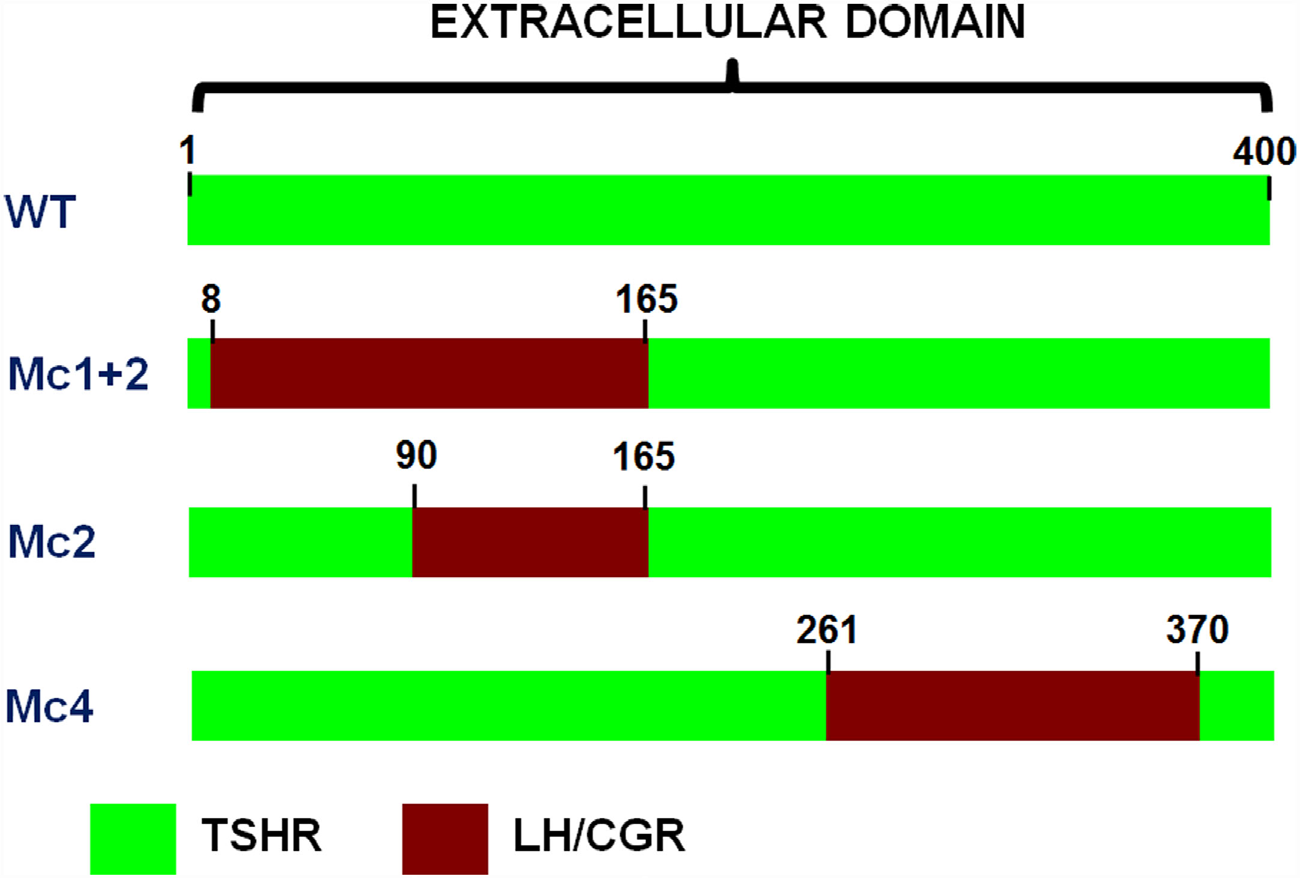
**Schematic representations of the extracellular regions of the three chimeric TSHR–LH/CGR mutants**. The Mc1 + 2, Mc2, and Mc4 chimera receptors. Green, human TSHR sequence (WT); red, homologous regions of the rat LH/CGR that were used to substitute for the deleted TSHR sequences. The numbers assigned to the amino acids are determined by counting from the methionine start site.

Moreover, the cloning of TSHR led to a series of studies that were mainly based on site-directed mutagenesis, deletion mutants, and the construction of receptor chimeras, which provided the pioneering achievements in the structure–function relationships of TSHR (31, 44, 45). The Kohn group was particularly involved in these studies, and in particular, in the construction of human TSHR–rat luteinizing hormone/chorionic gonadotropin receptor (TSHR–LH/CGR) chimeras (46), as these paved the way to the new TRAbs bioassays. A series of TSHR–LH/CGR chimeras were then constructed by replacing the homologous segments of the extracellular domain of the human TSHR with the corresponding segments of the rat LH/CGR, and these were used to identify receptor binding sites for TSH and TRAbs. Two chimeras were of particular interest and are known as the Mc1 + 2 and the Mc4 chimeras (Figure 3). The Mc1 + 2 chimera has a large portion of the N-terminal extracellular region of TSHR substituted (amino-acid residues 8–165), and it retains TSH binding and TSH stimulation of cAMP levels. However, the Mc1 + 2 chimera does not have the TSAbs activity, i.e., TSAbs cannot stimulate cAMP production or inhibit TSH binding to the chimera. However, its TBAbs binding affinity is maintained (Table 2). The Mc4 chimera has amino-acid residues 261–370 substituted, and it retains the ability for TSH and TSAbs binding and to still promote increased cAMP levels, whereas it no longer shows TBAbs binding (Table 2). These data suggested that the TRAbs that show different functional activities, i.e., TSAbs and TBAbs, have epitopes that are located in distinct regions of the extracellular domain of TSHR. More precisely, TSAbs are largely directed against the N-terminus region of TSHR, which includes amino-acid residues 8–165, whereas TBAbs mainly bind the C-terminal region, which includes amino-acid residues 261–370 (46, 47). Similar data were obtained simultaneously by the Rapoport group, who also demonstrated some degree of overlap between the epitopes for TSAbs and TBAbs (45, 48). Subsequent studies then showed significant overlap among these epitopes, while also describing more of their complex characteristics (49–51). However, the functional data described above induced Kohn to establish a new bioassay based on the use of these TSHR–LH/CGR chimeras to evaluate the clinical relevance of autoantibody heterogeneity in patients with Graves’ disease.

**Table 2:** **Summary of functional properties of TSHR–LH/CGR chimeras**.

Receptor/chimera	TSH binding	TSAb binding	TBAb binding
TSHR wild-type	Yes	Yes	Yes
Mc1 + 2	Yes	No	Yes
Mc2	Yes	No	Yes
Mc4	Yes	Yes	No

## The TSHR–LH/CGR Chimera Bioassay

The clinical use of the TRAbs bioassay based on the chimeric receptor was evaluated using CHO cells stably transfected with the rh-TSHR, the Mc1 + 2 TSHR–LH/CGR chimera described above, or the Mc2 chimera, in which residues 90–165 of the TSHR ectodomain were substituted (Figure 3). A preliminary study that was performed using purified IgG from 66 patients with Graves’ disease showed that although the TSAbs activities in the majority of patients was not detectable in the cells transfected with the Mc1 + 2 or Mc2 chimeras, in approximately 30% of the patients there were TSAbs that could activate these chimeric receptors (52). Therefore, these patients with Graves’ disease could be divided into two groups: a homogeneous group, with TSAbs that recognized only the N-terminal region of the TSHR ectodomain and did not activate the chimeric receptors, and a heterogeneous group, with TSAbs that interacted with the C-terminal region of the TSHR ectodomain and activated the chimeric receptors.

A very interesting observation came from the clinical correlation of these data. The heterogeneous group was more responsive to antithyroid therapies, which meant that the patients in this group were more likely to become euthyroid during treatment, and to do so more quickly. Moreover, a following study demonstrated that antithyroid drug therapies induced epitope heterogeneity, namely, during antithyroid treatment, about 50% of the patients with Graves’ disease who were initially negative in the chimera assay became positive (53). These data were confirmed in a larger study that was characterized by a longer follow-up, which indicated that heterogeneity of TSAbs is a good and independent marker for prediction of the clinical outcome of patients with Graves’ disease after antithyroid drug therapies (54).

## Improvement of the TRAbs Assay: New Generations of Immunoassays and Bioassays

In the late 1990s, a second generation of immunoassays was developed using moAbs against the C-terminus region of rh-TSHR or porcine TSHR. Plastic surfaces coated with these moAbs were used to immobilize TSHR, which was still able to bind TSH and TRAbs (55, 56).

This second-generation immunoassay, which is known as a “solid phase” assay, became the gold standard assay for TRAbs due to the high diagnostic accuracy and the use of a non-radioactive readout (15, 55–58). Indeed, a seminal study by Costagliola et al. (55) performed on 328 patients with Graves’ disease showed the high sensitivity and specificity of this assay (98.8 and 99.6%, respectively), with no differences between the radioactive or chemiluminescence readouts. The use of rh-TSHR or porcine TSHR did not affect the diagnostic accuracy of the assay (57). These data were confirmed by subsequent studies, as reported in a recent meta-analysis (15). Given this high diagnostic accuracy and the availability of a commercial kit, the “solid phase” assay became the most used assay for the detection of TRAbs.

At the same time, several researchers were involved in the generation of moAbs against TSHR characterized by TSAb activity (59–61). Due to the availability of rh-TSHR for both animal immunization and antibody screening, highly potent moAbs were obtained that were characterized by their higher affinities (reaching the order of nM concentrations), compared with the previous moAbs, where the concentrations used were in the order of μM or mM (24–26). The Davies group was particularly involved in these studies, and they used the moAbs as molecular probes to investigate further the structure–function relationships of TSHR and its interactions with TRAbs (1, 35, 62–65). A number of new insights came from these studies: (1) TSAbs and most TBAbs recognize conformational epitopes in the α subunit of TSHR (i.e., involving the first 316 amino acids), with these epitopes either distinct or overlapping; (2) some TBAbs bind epitopes in the N-terminus of the β subunit of TSHR; (3) N-TRAbs bind linear epitopes that are mainly in the cleavage region; (4) TSHR is present on the cell surface in both its cleaved and uncleaved forms, and it can exit as multimers; (5) As opposed to TSH, TSAbs do not accelerate the cleavage of TSHR, and this might explain the prolonged overstimulation of the thyroid gland in Graves’ patients; and (6) N-TRAbs can activate alternative signal pathways to the classical cAMP pathway. These data have been fundamental in the understanding the structure–function relationships of TSHR and its role in the pathogenesis of Graves’ disease. Furthermore, in 2003, this research on these moAbs led to the isolation and characterization of the human monoclonal TSAb M22 from lymphocytes of a patient with Graves’ disease (66).

A third-generation immunoassay was then developed based on the use of this M22 autoantibody (67). Indeed, given its high affinity binding to TSHR, the labeled M22 autoantibody was then used instead of labeled bovine TSH in inhibition assays, with significant improvements to the intra-assay coefficient of variation (15, 58). This new immunoassay became the preferred TRAbs assay due to its high diagnostic accuracy and feasibility. Indeed, the pooled sensitivity from all of the data reported in the literature is 97.4% (range, 95–99.6%), and the pooled specificity is 99.2% (range, 95–100%). Furthermore, the M22 assay is based on an ELISA method, and this is available also in a fully automated version (15, 68).

This availability of both stimulating (e.g., M22) and blocking human moAbs has also been useful for determination of the crystal structure of TSHR and its interactions with the TRAbs (49, 69). These studies confirmed the extensive overlap among the epitopes for TSAbs and TBAbs.

Concurrent with the development of the third-generation immunoassay, Kohn conceived the use of the Mc4 chimera (Figure 2) for a new bioassay. As indicated above, the Mc4 chimera retains the binding of TSH and TSAbs and the consequent activity but loses TBAbs binding (Table 2). Aside from arguments about different TSAbs and TBAbs epitopes, which as discussed above is a complex issue, several studies have provided the basis for the use of the Mc4 chimera, as reviewed by Lytton and Kahaly in 2010 (70). Further support for the use of the Mc4 chimera was provided by the finding that the shed A-subunit of the TSHR (spanning from approximately amino-acid residue 22–216), rather than the TSHR holoreceptor, is important for immunogenicity and for maturation affinity of TRAbs (71–74).

**Figure 4:**
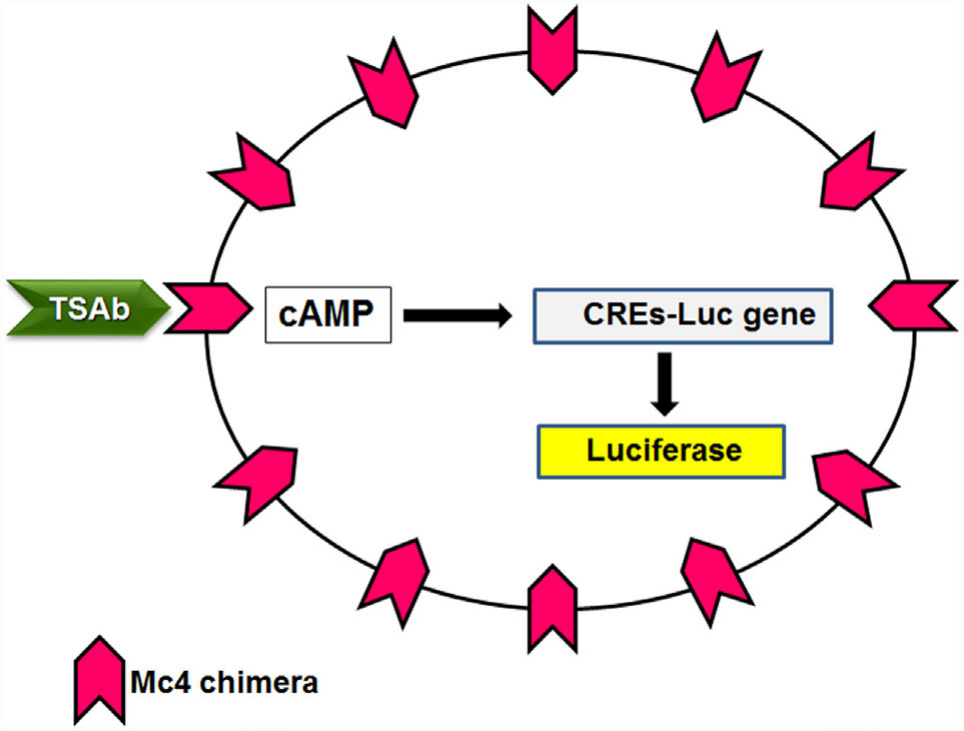
**Principle of the Mc4 bioassay**. Cell lines lacking TSHR are double transfected with a luciferase reporter gene under the transcriptional control of multiple cAMP-responsive elements (CREs-Luc) and the Mc4 chimeric receptor. TSAbs levels in patient sera are determined by measuring the increased production of luciferase.

This new bioassay is based on a chemiluminescent method, as described by Watson and colleagues (75), which uses cell lines that are stably transformed with a reporter plasmid that contains the firefly luciferase gene under the transcriptional control of multiple cAMP-responsive elements. These transformed cell lines were transfected with the Mc4 chimera (Figure 4) and were evaluated using sera from patients with Graves’ disease and other thyroid diseases, and normal subjects (76). The primary goal here was to create a bioassay that measured only TSAbs, without interference of the other TRAbs, and to have a clear cutoff between patients with Graves’ disease and the controls. For this purpose, the Mc4 assay was compared with a bioassay using wild-type TSHR and with a second-generation immunoassay. This study showed that the Mc4 assay has higher sensitivity and specificity (i.e., 100 and 98.5%, respectively) than the compared assays (Table 3). Furthermore, the Mc4 assay showed even higher sensitivity than the third-generation M22 immunoassay, although with a little less specificity (Table 3). The high diagnostic accuracy of the Mc4 assay can be attributed to the lack of interference by TBAbs and N-TRAbs. Indeed, contrary to what is observed with the conventional bioassay using wild-type TSHR, sera from patients with idiopathic myxedema, who have high TBAbs activity, did not inhibit the TSAb activity of the sera in the Mc4 bioassay (76).

**Table 3:** **Comparison among sensitivity and specificity of the main TRAbs assays**.

TRAbs assays	Sensitivity (%)	Specificity (%)
Mc4 bioassay	100^a^	98.5^a^
CHO–wtTSHR bioassay	97.3^a^	93.1^a^
“Solid phase” immunoassay	86.5^a^	97^a^
M22 ELISA assay	97.4^b^	99.2^b^

*^a^Data are from Ref. (76)*.

*^b^Data are from Ref. (15)*.

An important conclusion that came from this study of Giuliani et al. (76) was that, given its high diagnostic accuracy, the Mc4 assay can be used as a first-level test in the diagnosis of Graves’ disease. However, given the almost similar diagnostic accuracy and the better feasibility, the M22 immunoassay remains the preferred TRAbs assay worldwide to date. On the other hand, the specific detection of TSAbs without interference of other antibodies directed against TSHR makes the Mc4 assay potentially useful in the follow-up of patients with Graves’ disease. Indeed, one of the clinical problems of Graves’ disease is the high possibility of relapse within the first 2 years after withdrawal of medical therapy (at approximately 50%). Therefore, the results of a prospective study are of particular interest, where the Mc4 assay was shown to be a sensitive index of remission and relapse in patients with Graves’ disease (77). This study was performed in patients with Graves’ disease treated with antithyroid drugs (mainly methimazole) over a 5-year period, and it showed that the levels of TSAbs correlate with the clinical outcome of the disease. Furthermore, here, the Mc4 assay had high accuracy as a predictor of Graves’ disease prognosis, which was even better than the M22 third-generation immunoassay. A reasonable explanation for this is that the measure of only TSAbs instead of the whole spectrum of the TRAbs improves the prediction of the patient prognosis. Hence, the Mc4 assay might become a useful tool in identifying at an early stage those patients who will have no benefit from the medical therapy and to whom alternative therapeutic options can be offered. Indeed, failure to reduce TSAbs levels during medical therapy is a negative predictor of remission.

Retrospective studies have replicated the use of the Mc4 assay as a better indicator of the prognosis of patients with Graves’ disease than these other assays (77, 78). Furthermore, several studies have shown that the Mc4 bioassay strongly correlates with the indices of clinical activity and severity of Graves’ orbitopathy and has higher diagnostic accuracy than these other TRAbs assays (2, 70, 79–83). Indeed, a seminal study by Lytton et al. (79) showed that, compared with the second-generation immunoassays, the Mc4 assay had greater sensitivity (97 vs. 77%, respectively) and specificity (89 vs. 43%, respectively) for the detection of TRAbs in patients with Graves’ orbitopathy. Furthermore, this study demonstrated a strong correlation of TSAbs with the clinical activities of orbitopathy. Of interest, there was the observation that all patients whose sera were positive in the Mc4 assay and negative in the TRAbs immunoassay had severe orbitopathy, whereas those patients who tested negative with the Mc4 assay and positive with the TRAbs assay did not have active orbitopathy. These data confirm the superiority of the Mc4 assay in the detection of the subtypes of TRAbs that are directly involved in the pathogenesis of Graves’ disease, without the interference of the other subtypes, such as blocking or neutral TRAbs, that have little or no pathogenic role in the clinical manifestations of Graves’ disease. The studies that followed further confirmed these results and showed the usefulness of the Mc4 assay as a predictor of the clinical course of Graves’ orbitopathy (2, 80, 82).

A recent multicenter study (81) showed that the Mc4 assay is more sensitive than the third-generation immunoassay in diagnosing Graves’ disease in an untreated pediatric population. Moreover, as previously demonstrated in adult patients, the correlation of the Mc4 assay with the clinical activity and severity of Graves’ orbitopathy was higher than seen for the third-generation immunoassay in these pediatric patients.

Widespread use of this bioassay will be facilitated by the availability of the Mc4 assay as a commercial kit, which has a standardized protocol and good feasibility and reproducibility (84, 85). Indeed, using the commercial kit, the bioassay can be performed in less than 24 h (70), and the concentrations determined can be converted in IU/L, with the possibility to standardize the TSAbs levels across laboratories, which provide more accurate comparisons of TSAbs levels (84). Of note, recently, the Mc4 chimera has been used to develop a new *in vitro* assay by applying Bridge technology (86). In brief, this Bridge Assay uses two TSH chimeric receptors: the Mc4 chimera, which is used as a capture receptor that is anchored on a solid phase, to bind one arm of the autoantibody; and a chimeric receptor formed by the N-terminus (aminoacids 21–261) of TSHR fused with secretory alkaline phosphatase as a chemiluminescence monitor, which can bind the other arm of the autoantibody. Preliminary data show good sensitivity and specificity for this Bridge Assay (99.8 and 99.5%, respectively) (86).

## Conclusion

Thyroid-stimulating hormone receptor autoantibodies bioassays have several advantages in comparison to inhibition immunoassays. Bioassays can detect the functional heterogeneity of TRAbs in patients with Graves’ disease; i.e., the simultaneous presence in the same patient of TSAbs, TBAbs, and/or N-TRAbs. This has clinical implications, because the switching from TSAbs to TBAbs is responsible for the evolution toward hypothyroidism in a small percentage of patients with Graves’ disease. Moreover, a selective decrease in TSAbs is a positive prognostic feature for patient remission. Evaluation of TSAbs is also important in pregnant woman with Graves’ disease, to estimate the risk of fetal/neonatal thyrotoxicosis due to TRAbs transfer. Finally, the monitoring of the switch from TSAbs to TBAbs, and *vice versa*, is very useful in patients with alternate episodes of hyperthyroidism and hypothyroidism.

In the past 60 years, TRAbs bioassays have evolved from cumbersome and time-consuming procedures to genetically engineered cell-based assays that are characterized by good feasibility and rapid operating times, and that are also available as commercial kits. The role that Kohn had in this process through all of these years was fundamental. Indeed, initially, the use of the FRTL-5 bioassay, and then the later generation of the Mc4 bioassay, led to striking progress in both the knowledge of the functional features of TRAbs and the clinical application of TRAbs bioassays. Kohn perceived the advantages that the use of the Mc4 chimera would bring in diagnostic accuracy and prognostic evaluation for patients with Graves’ disease. He devoted himself to the improvement of the feasibility of the Mc4 bioassay to promote its use in clinical practice (85). The availability of the Mc4 bioassay as a commercial kit is now spreading the use of this assay worldwide. We believe that the improved feasibility of the Mc4 assay, together with its high diagnostic accuracy and prognostic use, will now make the Mc4 assay the preferred assay for clinical evaluation of patients with Graves’ disease.

## Author Contributions

CG: substantial contributions to the conception and design of the work; drafting the work; final approval of the version to be published; and agreement to be accountable for all aspects of the work in ensuring that questions related to the accuracy or integrity of any part of the work are appropriately investigated and resolved. MS: substantial contributions to the conception of the work; revising the work critically for important intellectual content; final approval of the version to be published; and agreement to be accountable for all aspects of the work in ensuring that questions related to the accuracy or integrity of any part of the work are appropriately investigated and resolved. IB: substantial contributions to the design of the work; revising the work critically for important intellectual content; final approval of the version to be published; and agreement to be accountable for all aspects of the work in ensuring that questions related to the accuracy or integrity of any part of the work are appropriately investigated and resolved. GN: substantial contributions to the conception of the work; revising it critically for intellectual content; final approval of the version to be published; and agreement to be accountable for all aspects of the work.

## Conflict of Interest Statement

The authors declare that the research was conducted in the absence of any commercial or financial relationships that could be construed as a potential conflict of interest. The reviewer TA and handling Editor declared their shared affiliation, and the handling Editor states that the process nevertheless met the standards of a fair and objective review.

## References

[1] Michalek K Morshed SA Latif R Davies TF. TSH receptor autoantibodies. Autoimmun Rev (2009) 9:113–6. 10.1016/j.autrev.2009.03.012 19332151 PMC3753030

[2] Jang SY Shin DY Lee EJ Lee SY Yoon JS. Relevance of TSH-receptor antibody levels in predicting disease course in Graves’ orbitopathy: comparison of the third-generation TBII assay and Mc4-TSI bioassay. Eye (Lond) (2013) 27:964–71. 10.1038/eye.2013.120 23743527 PMC3740315

[3] Bartalena L Burch HB Burman KD Kahaly GJ A 2013 European survey of clinical practice patterns in the management of Graves’ disease. Clin Endocrinol (2016) 84:115–20. 10.1111/cen.12688 25581877

[4] Giuliani C Monaco F Adult primary hypothyroidism. In: Monaco F, editor. Thyroid Diseases. Boca Raton, FL: CRC Press (2012). p. 171–94.

[5] Adams DD Purves HD Abnormal responses in the assay of thyrotropin. Proc Univ Otago Med Sch (1956) 34:11–5.

[6] Adams DD The presence of an abnormal thyroid-stimulating hormone in the serum of some thyrotoxic patients. J Clin Endocrinol Metab (1958) 18:699–712. 10.1210/jcem-18-7-699 13549548

[7] Adams DD Bioassay of long-acting thyroid stimulator (LATS); the dose-response relationship. J Clin Endocrinol Metab (1961) 21:799–805. 10.1210/jcem-21-7-799 13681365

[8] Kriss JP Pleshakov V Chien JR Isolation and identification of the long-acting thyroid stimulator and its relation to hyperthyroidism and circumscribed pretibial myxedema. J Clin Endocrinol Metab (1964) 24:1005–28. 10.1210/jcem-24-10-1005 14228525

[9] Kriss JP Inactivation of long-acting thyroid stimulator (LATS) by anti-kappa and anti-lamba antisera. J Clin Endocrinol Metab (1968) 28:1440–4. 10.1210/jcem-28-10-1440 5681638

[10] Volpé R The immunologic basis of Graves’s disease. N Engl J Med (1972) 287:463–4. 10.1056/NEJM197208312870910 5068098

[11] McKenzie JM Review: pathogenesis of Graves’ disease: role of the long-acting thyroid stimulator. J Clin Endocrinol Metab (1965) 25:424–31. 10.1210/jcem-25-3-424 14268493

[12] Mehdi SQ Nussey SS A radio-ligand receptor assay for the long-acting thyroid stimulator. Inhibition by the long-acting thyroid stimulator of the binding of radioiodinated thyroid stimulating hormone to human thyroid membranes. Biochem J (1975) 145:105–11. 10.1042/bj1450105 1191248 PMC1165191

[13] Shewring G Rees Smith B. An improved radioreceptor assay for TSH receptor antibodies. Clin Endocrinol (1982) 17:409–17. 10.1111/j.1365-2265.1982.tb01607.x 6291808

[14] Southgate K Creagh FM Teece M Kingswood C Rees Smith B. A receptor assay for the measurement of TSH receptor antibodies in unextracted serum. Clin Endocrinol (1984) 20:539–48. 10.1111/j.1365-2265.1984.tb00102.x 6086180

[15] Tozzoli R Bagnasco M Giavarina D Bizzarro N TSH receptor autoantibody immunoassay in patients with Graves’ disease: improvement of diagnostic accuracy over different generations of methods, systematic review and meta-analysis. Autoimmun Rev (2012) 12:107–13. 10.1016/j.autrev.2012.07.003 22776786

[16] Toccafondi RS Aterini S Medici MA Rotella CM Tanini A Zonefrati R Thyroid-stimulating antibody (TSAb) detected in sera of Graves’ patients using human thyroid cell cultures. Clin Exp Immunol (1980) 40:532–9. 6251989 PMC1538941

[17] Ambesi-Impiombato FS Parks LAM Coon HG. Culture of hormone-dependent functional epithelial cells from rat thyroids. Proc Natl Acad Sci U S A (1980) 77:3455–9. 10.1073/pnas.77.6.3455 6106191 PMC349635

[18] Vitti P Valente WA Ambesi-Impiombato FS Fenzi GF Pinchera A Kohn LD Graves’ IgG stimulation of continuously cultured rat thyroid cells: a sensitive and potentially useful clinical assay. J Endocrinol Invest (1982) 5:179–82. 10.1007/BF03349476 6286749

[19] Vitti P Rotella CM Valente WA Cohen J Aloj SM Laccetti P Characterization of the optimal stimulatory effects of Graves’ monoclonal and serum Immunoglobulin G on adenosine 3′,5′-monophosphate production in FRTL-5 thyroid cells: a potential clinical assay. J Clin Endocrinol Metab (1983) 57:782–91. 10.1210/jcem-57-4-782 6136523

[20] McKenzie JM Zakarija M. Clinical review 3. The clinical use of thyrotropin receptor antibody measurements. J Clin Endocrinol Metab (1989) 69:1093–6. 10.1210/jcem-69-6-1093 2685006

[21] Morris JC Hay ID Nelson RE Jiang NS. Clinical utility of thyrotropin-receptor antibody assays: comparison of radioreceptor and bioassay methods. Mayo Clin Proc (1988) 63:707–17. 10.1016/S0025-6196(12)65533-5 2898572

[22] Kohn LD Valente WA Grollman EF Aloj SM Vitti P, Inventors; Interthyr Research Foundation Inc., Assignee. Clinical Determination and/or Quantification of Thyrotropin and a Variety of Thyroid Stimulatory or Inhibitory Factors Performed In Vitro with an Improved Thyroid Cell Line, FRTL-5. United States patent US 4609622 (1986).

[23] Vitti P Chiovato L Lopez G Lombardi A Santini F Mammoli C Measurement of TSAb directly in serum using FRTL-5 cells. J Endocrinol Invest (1988) 11:313–7. 10.1007/BF03350157 2900851

[24] Yavin E Yavin Z Schneider MD Kohn LD Monoclonal antibodies to the thyrotropin receptor: implications for receptor structure and the action of autoantibodies in Graves’ disease. Proc Natl Acad Sci U S A (1981) 78:3180–4. 10.1073/pnas.78.5.3180 6265939 PMC319524

[25] Valente WA Vitti P Yavin Z Yavin E Rotella CM Grollman EF Monoclonal antibodies to the thyrotropin receptor: stimulating and blocking antibodies derived from the lymphocytes of patients with Graves’ disease. Proc Natl Acad Sci U S A (1982) 79:6680–4. 10.1073/pnas.79.21.6680 6292912 PMC347192

[26] Valente WA Yavin Z Yavin E Grollman EF Schneider M Rotella CM Monoclonal antibodies to the thyrotropin receptor: the identification of blocking and stimulating antibodies. J Endocrinol Invest (1982) 5:293–301. 10.1007/BF03350517 6296219

[27] Orgiazzi J Williams DE Chopra IJ Solomon DH Human thyroid adenyl cyclase-stimulating activity in immunoglobulin G of patients with Graves’ disease. J Clin Endocrinol Metab (1976) 42:341–54. 10.1210/jcem-42-2-341 946604

[28] Irvine WJ Lamberg BA Cullen DR Gordin R. Primary hypothyroidism preceding thyrotoxicosis: a report of 2 cases and a review of the literature. J Clin Lab Immunol (1979) 2:349–52. 583430

[29] Valente WA Vitti P Rotella CM Vaughan MM Aloj SM Grollman EF Antibodies that promote thyroid growth. A distinct population of thyroid-stimulating autoantibodies. N Engl J Med (1983) 309:1028–34. 10.1056/NEJM198310273091705 6137770

[30] Tahara K Grollman EF Saji M Kohn LD. Regulation of prostaglandin synthesis by thyrotropin, insulin or insulin-like growth factor-I, and serum in FRTL-5 rat thyroid cells. J Biol Chem (1991) 266:440–8. 1845972

[31] Kohn LD Shimura H Shimura Y Hidaka A Giuliani C Napolitano G The thyrotropin receptor. Vitam Horm (1995) 50:287–384. 10.1016/S0083-6729(08)60658-5 7709602

[32] Di Cerbo A Di Girolamo M Guardabasso V De Filippis V Corda D Immunoglobulins from Graves’ patients stimulate phospholipase-A2 in FRTL-5 thyroid cells. J Clin Endocrinol Metab (1992) 74:585–92. 10.1210/jc.74.3.585 1310998

[33] Di Cerbo A Di Paola R Menzaghi C De Filippis V Tahara K Corda D Graves’ immunoglobulins activate phospholipase-A2 by recognizing specific epitopes on thyrotropin receptor. J Clin Endocrinol Metab (1999) 84:3283–92. 10.1210/jcem.84.9.5967 10487700

[34] Morshed SA Latif R Davies TF. Characterization of thyrotropin receptor antibody-induced signaling cascades. Endocrinology (2009) 150:519–29. 10.1210/en.2008-0878 18719020 PMC2630889

[35] Morshed SA Ando T Latif R Davies TF Neutral antibodies to the TSH receptor are present in Graves’ disease and regulate selective signaling cascades. Endocrinology (2010) 151:5537–49. 10.1210/en.2010-0424 20844004 PMC2954721

[36] Ihara Y Kanda Y Seo M Watanabe Y Akamizu T Tanaka Y Growth stimulating antibody, as another predisposing factor of Graves’ disease (GD): analysis using monoclonal TSH receptor antibodies derived from patients with GD. Endocr J (2012) 59:571–7. 10.1507/endocrj.EJ11-0348 22510947

[37] Parmentier M Libert F Maenhaut C Lefort Gerard C Perret J Van Sande J Molecular cloning of the thyrotropin receptor. Science (1989) 246:1620–2. 10.1126/science.2556796 2556796

[38] Nagayama Y Kaufman KD Seto P Rapoport B. Molecular cloning, sequence and functional expression of the cDNA for the human thyrotropin receptor. Biochem Biophys Res Commun (1989) 165:1184–90. 10.1016/0006-291X(89)92727-7 2558651

[39] Akamizu T Ikuyama S Saji M Kosugi S Kozak C McBride OW Cloning, chromosomal assignment, and regulation of the rat thyrotropin receptor: expression of the gene is regulated by thyrotropin, agents that increase cAMP levels, and thyroid autoantibodies. Proc Natl Acad Sci U S A (1990) 87:5677–81. 10.1073/pnas.87.15.5677 1696008 PMC54390

[40] Ludgate M Perret J Parmentier M Gerard C Libert F Dumont JE Use of recombinant human thyrotropin receptor (TSH-R) expressed in mammalian cell lines to assay TSH-R autoantibodies. Mol Cell Endocrinol (1991) 73:R13–8. 10.1016/0303-7207(90)90050-I 1981364

[41] Filetti S Foti D Costante G Rapoport B. Recombinant human thyrotropin (TSH) receptor in a radioreceptor assay for the measurement of TSH receptor autoantibodies. J Clin Endocrinol Metab (1991) 72:1096–101. 10.1210/jcem-72-5-1096 2022709

[42] Vitti P Elisei R Tonacchera M Chiovato L Mancusi F Rago T Detection of thyroid-stimulating antibody using Chinese hamster ovary cells transfected with cloned human thyrotropin receptor. J Clin Endocrinol Metab (1993) 76:499–503. 10.1210/jcem.76.2.8094393 8094393

[43] Kim MR Faiman C Hoogwerf BJ Gupta MK Thyroid-stimulating antibody assay using a human thyrotropin receptor transfected cell line: relationship to clinical features of Graves’ disease. Endocr Pract (1997) 3:337–43. 10.4158/EP.3.6.337 15251770

[44] Kohn LD Giuliani C Montani V Napolitano G Ohmori M Ohta M Antireceptor immunity. In: Rayner DC Champion BR, editors. Thyroid Autoimmunity. Austin, TX: RG Landes Company (1995). p. 115–70.

[45] Rapoport B Chazenbalk GD Jaume JC McLachlan SM The thyrotropin (TSH) receptor: interaction with TSH and autoantibodies. Endocr Rev (1998) 19:673–716. 10.1210/er.19.6.673 9861544

[46] Tahara K Ban T Minegishi T Kohn LD. Immunoglobulins from Graves’ disease patients interact with different sites on TSH receptor/LH-CG receptor chimeras than either TSH or immunoglobulins from idiopathic myxedema patients. Biochem Biophys Res Commun (1991) 179:70–7. 10.1016/0006-291X(91)91335-A 1883391

[47] Tahara K Ishikawa N Yamamoto K Hirai A Ito K Tamura Y Epitopes for thyroid stimulating and blocking autoantibodies on the extracellular domain of the human thyrotropin receptor. Thyroid (1997) 7:867–77. 10.1089/thy.1997.7.867 9459630

[48] Nagayama Y Wadsworth HL Russo D Chazenbalk GD Rapoport B. Binding domains of stimulatory and inhibitory thyrotropin (TSH) receptor autoantibodies determined with chimeric TSH-lutropin/chorionic gonadotropin receptors. J Clin Invest (1991) 88:336–40. 10.1172/JCI115297 1711544 PMC296038

[49] Nunez Miguel R Sanders J Sanders P Young S Clark J Kabelis K Similarities and differences in interactions of thyroid stimulating and blocking autoantibodies with the TSH receptor. J Mol Endocrinol (2012) 49:137–51. 10.1530/JME-12-0040 22829655

[50] Latif R Teixeira A Michalek K Ali MR Schlesinger M Baliram R Antibody protection reveals extended epitopes on the human TSH receptor. PLoS One (2012) 7(6):e44669. 10.1371/journal.pone.0044669 22957097 PMC3434159

[51] Davies TF Latif R. Targeting the thyroid-stimulating hormone receptor with small molecule ligands and antibodies. Expert Opin Ther Targets (2015) 19:835–47. 10.1517/14728222.2015.1018181 25768836 PMC4484771

[52] Kim WB Cho BY Park HY Lee HK Kohn LD Tahara K Epitopes for thyroid-stimulating antibodies in Graves’ sera: a possible link of heterogeneity to differences in response to antithyroid drug treatment. J Clin Endocrinol Metab (1996) 81:1758–67. 10.1210/jc.81.5.1758 8626830

[53] Kim WB Chung HK Lee HK Kohn LD Tahara K Cho BY Changes in epitopes for thyroid-stimulating antibodies in Graves’ disease sera during treatment of hyperthyroidism: therapeutic implications. J Clin Endocrinol Metab (1997) 82:1953–9. 10.1210/jc.82.6.1953 9177413

[54] Kim TY Park YJ Park DJ Chung HK Kim WB Kohn LD Epitopes heterogeneity of thyroid-stimulating antibodies predicts long-term outcome in Graves’ patients treated with antithyroid drugs. J Clin Endocrinol Metab (2003) 88:117–24. 10.1210/jc.2002-020389 12519839

[55] Costagliola S Morgenthaler NG Hoermann R Badenhoop K Struck J Freitag D Second generation assay for thyrotropin receptor antibodies has superior diagnostic sensitivity for Graves’ disease. J Clin Endocrinol Metab (1999) 84:90–7. 10.1210/jc.84.1.90 9920067

[56] Sanders J Oda Y Roberts S Kiddie A Richards T Bolton J The interaction of TSH receptor autoantibodies with ^125^I-labelled TSH receptor. J Clin Endocrinol Metab (1999) 84:3797–802. 10.1210/jcem.84.10.6071 10523032

[57] Kamijo K TSH-receptor antibody measurement in patients with various thyrotoxicosis and Hashimoto’s thyroiditis: a comparison of two two-step assays, coated plate ELISA using porcine TSH-receptor and coated tube radioassay using human recombinant TSH-receptor. Endocr J (2003) 50:113–6. 10.1507/endocrj.50.113 12733717

[58] Barbesino G Tomer Y Clinical utility of TSH receptor antibodies. J Clin Endocrinol Metab (2013) 98:2247–55. 10.1210/jc.2012-4309 23539719 PMC3667257

[59] Ando T Latif R Pritsker A Moran T Nagayama Y Davies TF. A monoclonal thyroid-stimulating antibody. J Clin Invest (2002) 110:1667–74. 10.1172/JCI0216991 12464672 PMC151640

[60] Costagliola S Franssen JDF Bonomi M Urizar E Willnich M Bergmann A Generation of a mouse monoclonal TSH receptor antibody with stimulating activity. Biochem Biophys Res Commun (2002) 299:891–6. 10.1016/S0006-291X(02)02762-6 12470663

[61] Sanders J Jeffreys J Depraetere H Richards T Evans M Kiddie A Thyroid-stimulating monoclonal antibodies. Thyroid (2002) 12:1043–50. 10.1089/105072502321085135 12593717

[62] Ando T Latif R Daniel S Eguchi K Davies TF. Dissecting linear and conformational epitopes on the native thyrotropin receptor. Endocrinology (2004) 145:5185–93. 10.1210/en.2004-0789 15297445

[63] Latif R Ando T Davies TF. Monomerization as a prerequisite for intramolecular cleavage and shedding of the thyrotropin receptor. Endocrinology (2004) 145:5580–8. 10.1210/en.2004-0797 15319351

[64] Ando T Davies TF Monoclonal antibodies to the thyrotropin receptor. Clin Dev Immunol (2005) 12:137–43. 10.1080/17402520500078238 16050145 PMC2270726

[65] Ando T Latif R Davies TF. Antibody-induced modulation of TSH receptor post-translational processing. J Endocrinol (2007) 195:179–86. 10.1677/JOE-07-0058 17911409

[66] Sanders J Evans M Premawardhana LDKE Depraetere H Jeffreys J Richards T Human monoclonal stimulating autoantibody. Lancet (2003) 362:126–8. 10.1016/S0140-6736(03)13866-4 12867115

[67] Rees Smith B Bolton J Young S Collyer A Weeden A Bradbury J A new assay for thyrotropin receptor autoantibodies. Thyroid (2004) 14:830–5. 10.1089/1050725042451248 15588379

[68] Zophel K Roggenbuck D von Landenberg P Wunderlich G Gruning T Kotzerke J TSH receptor antibody (TRAb) assays based on the human monoclonal autoantibody M22 are more sensitive than bovine TSH based assays. Horm Metab Res (2010) 42:65–9. 10.1055/s-0029-1241196 19830651

[69] Furmaniak J Sanders J Nunez Miguel R Rees Smith B. Mechanisms of action of TSHR autoantibodies. Horm Metab Res (2015) 47:735–52. 10.1055/s-0035-1559648 26361260

[70] Lytton SD Kahaly GJ. Bioassays for TSH-receptor autoantibodies: an update. Autoimmun Rev (2010) 10:116–22. 10.1016/j.autrev.2010.08.018 20807591

[71] Chanzenbalk GD Pichurin P Chen CR Latrofa F Johnstone AP McLachlan SM Thyroid-stimulating antibodies in Graves’ diseases preferentially recognize the free subunit, not the holoreceptor. J Clin Invest (2002) 110:209–17. 10.1172/JCI0215745 12122113 PMC151066

[72] Chen CR Pichurin P Nagayama Y Latrofa F Rapoport B McLachlan SM The thyrotropin receptor autoantigen in Graves is the culprit as well as the victim. J Clin Invest (2003) 111:1897–904. 10.1172/JCI200317069 12813025 PMC161420

[73] Mizutori Y Chen CR Latrofa F McLachlan SM Rapoport B Evidence that shed TSH receptor A-subunits drive affinity maturation of autoantibodies causing Graves’ disease. J Clin Endocrinol Metab (2009) 94:927–35. 10.1210/jc.2008-2134 19066298 PMC2681282

[74] Rapoport B McLachlan SM. TSH receptor cleavage into subunits and shedding of the A-subunit; a molecular and clinical perspective. Endocr Rev (2016) 37:113–34. 10.1210/er.2015-1098 26799472 PMC4823380

[75] Watson PF Ajjan RA Phipps J Metcalfe R Weetman AP A new chemiluminescent assay for the rapid detection of thyroid stimulating antibodies in Graves’ disease. Clin Endocrinol (1998) 49:577–81. 10.1046/j.1365-2265.1998.00619.x 10197071

[76] Giuliani C Cerrone D Harii N Thornton M Kohn LD Dagia NM A TSHr-LH/CGr chimera that measures functional TSAb in Graves’ disease. J Clin Endocrinol Metab (2012) 97:E1106–15. 10.1210/jc.2011-2893 22496495

[77] Giuliani C Cerrone D Harii N Thornton M Kohn LD Dagia NM A TSHr-LH/CGr chimera that measures functional thyroid-stimulating autoantibodies (TSAb) can predict remission or recurrence in Graves’ patients undergoing antithyroid drug (ATD) treatment. J Clin Endocrinol Metab (2012) 97:E1080–7. 10.1210/jc.2011-2897 22492869

[78] Hwang S Shin DY Song MK Lee EJ High cut-off value of a chimeric TSH receptor (Mc4)-based bioassay may improve prediction of relapse in Graves’ disease for 12 months. Endocrine (2015) 48:89–95. 10.1007/s12020-014-0325-8 24968734

[79] Lytton SD Ponto KA Kanitz M Matheis N Kohn LD Kahaly GJ A novel thyroid stimulating immunoglobulin bioassay is a functional indicator of activity and severity of Graves’ orbitopathy. J Clin Endocrinol Metab (2010) 95:2123–31. 10.1210/jc.2009-2470 20237164

[80] Jang SY Shin DY Lee EJ Yoon JS Clinical characteristics of Graves’ orbitopathy in patients showing discrepancy between levels from TBII assay and TSI bioassay. Clin Endocrinol (2014) 80:591–7. 10.1111/cen.12318 24033537

[81] Diana T Brown RS Bossowski A Segni M Niedziela M Koning J Clinical relevance of thyroid-stimulating autoantibodies in pediatric Graves’ disease. A multicenter study. J Clin Endocrinol Metab (2014) 99:1648–55. 10.1210/jc.2013-4026 24517152

[82] Ponto KA Kanitz M Olivo PD Pitz S Pfeiffer N Kahaly GJ Clinical relevance of thyroid-stimulating immunoglobulins in Graves’ ophthalmopathy. Ophthalmology (2011) 118:2279–85. 10.1016/j.ophtha.2011.03.030 21684605

[83] Kamijo K Murayama H Uzu T Togashi K Kahaly GJ A novel bioreporter assay for thyrotropin receptor antibodies using a chimeric thyrotropin receptor (Mc4) is more useful in differentiation of Graves’ disease from painless thyroiditis than conventional thyrotropin-stimulating antibody assay using porcine thyroid cells. Thyroid (2010) 20:851–6. 10.1089/thy.2010.0059 20615137

[84] Diana T Kanitz M Lehman M Li Y Olivo PD Kahaly GJ. Standardization of a bioassay for thyrotropin receptor stimulating autoantibodies. Thyroid (2015) 25:169–75. 10.1089/thy.2014.0346 25317659

[85] Kohn LD Brown J Scholl D Li Y Napolitano G, Inventors; Diagnostic Hybrids Inc., Assignee. Sensitive and Rapid Methods of Using Chimeric Receptors to Identify Autoimmune Disease and Assess Disease Severity. United States patent US 8563257B2 (2013).

[86] Frank CU Braeth S Dietrich JW Wanjura D Loos U Bridge technology with TSH receptor chimera for sensitive direct detection of TSH receptor antibodies causing Graves’ disease: analytical and clinical evaluation. Horm Metab Res (2015) 47:880–8. 10.1055/s-0035-1554662 26079838

